# Application of metagenomics sequencing to diagnose paralytic rabies with stroke-like onset: a case report

**DOI:** 10.3389/fmed.2025.1639262

**Published:** 2025-08-26

**Authors:** Baoquan Lin, Dan Zhong, Lu Qin, Qianqian Liu, Liya Wu, Bo Wang, Kuliang Wang, Xianfu Lu, Shan Deng, Liya Pan

**Affiliations:** Department of Neurology, The Fourth Affiliated Hospital of Guangxi Medical University, Liuzhou, China

**Keywords:** stroke, paralytic rabies, cerebrospinal fluid, whole genome sequencing, zoonotic infectious disease

## Abstract

**Background:**

Rabies is an acute zoonotic infectious disease caused by infection with a virus of the genus Lyssavirus. We report a case of paralytic rabies with a stroke-like onset, which was diagnosed using metagenomics next-generation sequencing (mNGS).

**Case presentation:**

A 58-year-old man was admitted to the hospital with “numbness and weakness in the right upper extremity for 2 days, aggravated for 1 day.” Twenty-five days before his admission, the patient was bitten on the back of right hand by an unvaccinated domestic dog, resulting in a penetrating injury, classified as grade III according to the rabies exposure classification method. Following admission, the patient exhibited rapidly progressive stroke symptoms, and on the second day, he suffered a sudden respiratory arrest accompanied by a weakened heartbeat and a decreased heart rate. He was treated with emergency tracheal intubation, cardiopulmonary resuscitation, and dehydration to lower cranial pressure.

**Results:**

The patient’s condition deteriorated rapidly after admission. A lumbar puncture was conducted on the morning of the second day of admission, and cerebrospinal fluid (CSF) was sent to Weiyuan Genetic Laboratories (Guangzhou, China) for rabies virus identification. The patient died on the third day of admission. Pathogen capture macro-genomics was performed on CSF using an Illumina NextSeq second-generation sequencer, and nine rabies virus sequences, which shared more than 99% nucleotide homology with the genome sequence of the rabies virus Rabies lyssavirus (NCBI accession no. MN175989.1), were detected. The Q30 ratio of this test was 98.3%.

**Conclusion:**

Compared to polymerase chain reaction (PCR) and direct fluorescent antibody (DFA) test, mNGS shortens the diagnostic window and improves sensitivity to low-virus or seronegative manifestations by simultaneously capturing and sequencing the entire pathogen genome. The mNGS technology can effectively aid in the diagnosis of paralytic rabies.

## 1 Introduction

Once rabies develops, the fatality rate is almost 100%, and there is still a lack of effective treatment (1, 2). It is estimated that rabies causes 50,000 to 75,000 deaths worldwide each year (3). Rabies is a zoonotic disease that is prevalent worldwide with the highest incidence in Asia and Africa. The estimated annual number of human cases is about 75,000 (4). In China, the incidence rate of rabies has shown a consistent decline over the past decade or so, attributed to the continuous improvement of relevant laws and regulations improved outbreak monitoring systems, increased accessibility and standardization of post-exposure rabies management and the persistent strengthening of dog management and stray animal rescue efforts (5, 6).

Rabies is an acute zoonotic infectious disease caused by infection with a virus of the genus Lyssavirus. According to the most recent classification by the International Committee on Taxonomy of Viruses (ICTV) in 2021, there are 17 species of lyssaviruses, which are categorized into three genetic lineages based on their genetic makeup and serological cross-reactivity. Among them, the rabies virus (RABV) belonging to genetic lineage I, serves as the representative species of the genus. RABV exhibits a high degree of neurotropism and is the primary pathogen causing rabies in humans. Current rabies vaccines are usually only effective against genetic lineage I lyssaviruses.

The clinical course of rabies can be divided into three phases: prodromal, excitatory, and paralytic. The development of rabies is a continuous process, and these stages are not distinctly separable. Based on clinical features, rabies can be divided into manic and paralytic types, with the manic type representing more than 80% and the paralytic type less than 20%. The diagnosis of manic rabies is not difficult based on the history of exposure and typical rabies syndromes. However, there are still some atypical cases, especially paralytic rabies, which can present in a variety of forms at onset and may be easily misdiagnosed in the early stages. We report a case of stroke-like onset paralytic rabies in which the final cerebrospinal fluid (CSF) was sent to the Weiyuan Genetics Laboratory for whole genome sequencing to identify the rabies virus. Pathogen capture metagenomics testing of CSF using a second-generation sequencer, Illumina NextSeq, successfully detected the sequences of the rabies virus, leading to the diagnosis of paralytic rabies.

## 2 Case presentation

**Figure 1:**
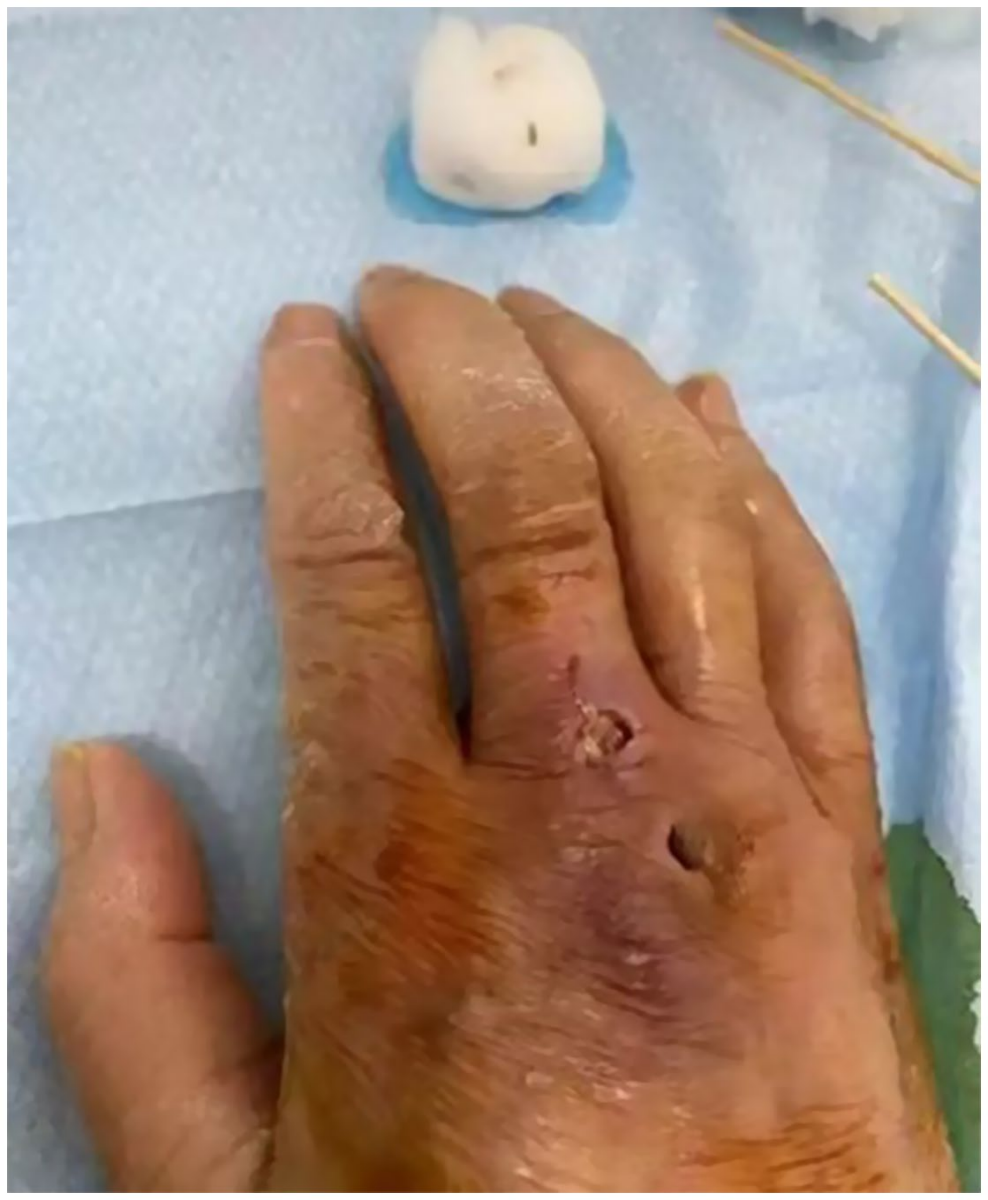
The patient was bitten by a dog on the back of his right hand.

**Figure 2:**
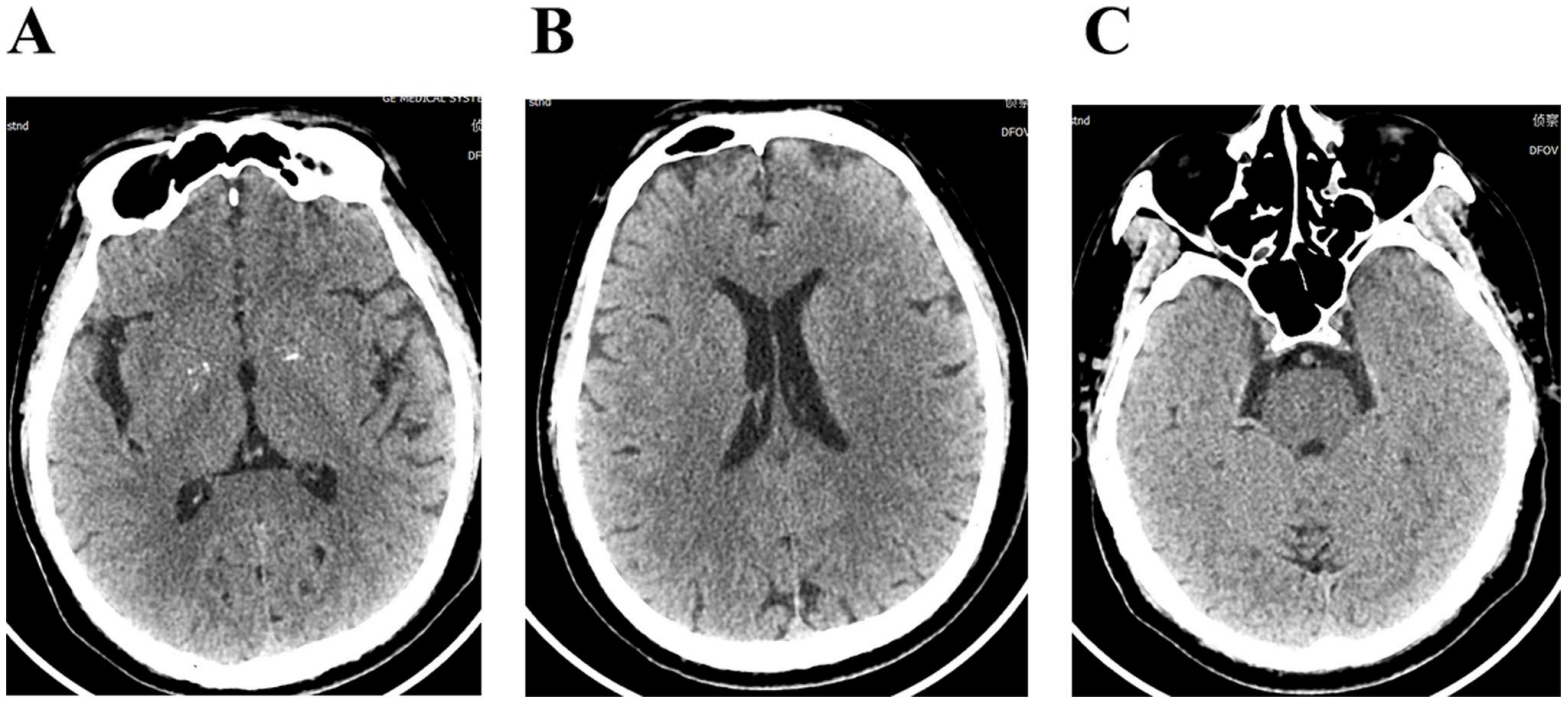
**(A–C)** Cranial computerized tomography showed no significant abnormalities.

The patient, a 58-year-old male farmer, was admitted to the hospital on November 08, 2023, due to “numbness and weakness of the right upper limb for 2 days, aggravated for 1 day.” On October 14, 2023, he was bitten by an unregulated, unvaccinated domestic dog on the back of the right hand, resulting in penetrating injuries and bleeding (Figure 1). The wounds were simply washed and disinfected, and the treatment of wounds was not standardized. There was no passive immunization against rabies, and only the freeze-dried rabies vaccine (Liaoning Chengda Vero) was routinely administered. According to the “2-1-1” immunization program, two doses of rabies vaccine were injected on the day 0 (1 dose for each of the deltoid muscles of the left and right upper arms), followed by one dose on the day 7 and 21, respectively. The classification of rabies exposure based on the mode of exposure and degree of exposure is categorized as class III exposure. The patient displayed with weakness and numbness in the distal right upper extremity starting 23 days after the canine injury. The weakness primarily affected the distal of the right upper limb, initially manifesting as mild characterized by an inability to perform fine motor movements, and was accompanied by neck and shoulder pain, nausea, vomiting. On the day 24, the weakness and numbness in the distal right upper limb progressively worsened, accompanied by fever with a maximum temperature of 38.8 °C. There were no signs of slurred vision, visual field defects, or gibberish. Furthermore, there were no indications of slurred speech, difficulties in swallowing, or choking on water. The patient was admitted to the outpatient clinic and later to the hospital for “acute cerebral infarction” after an emergency computerized tomography (CT) scan that showed no abnormalities (Figure 2). There was a denial of any recent infections. The patient also denied any previous cerebrovascular disease risk factors such as hypertension, diabetes mellitus, coronary artery disease, hyperlipidemia, and hyperuricemia; however, there was a history of epilepsy. The patient received three complete doses of COVID-19 vaccine beginning June 1, 2021.

**Figure 3:**
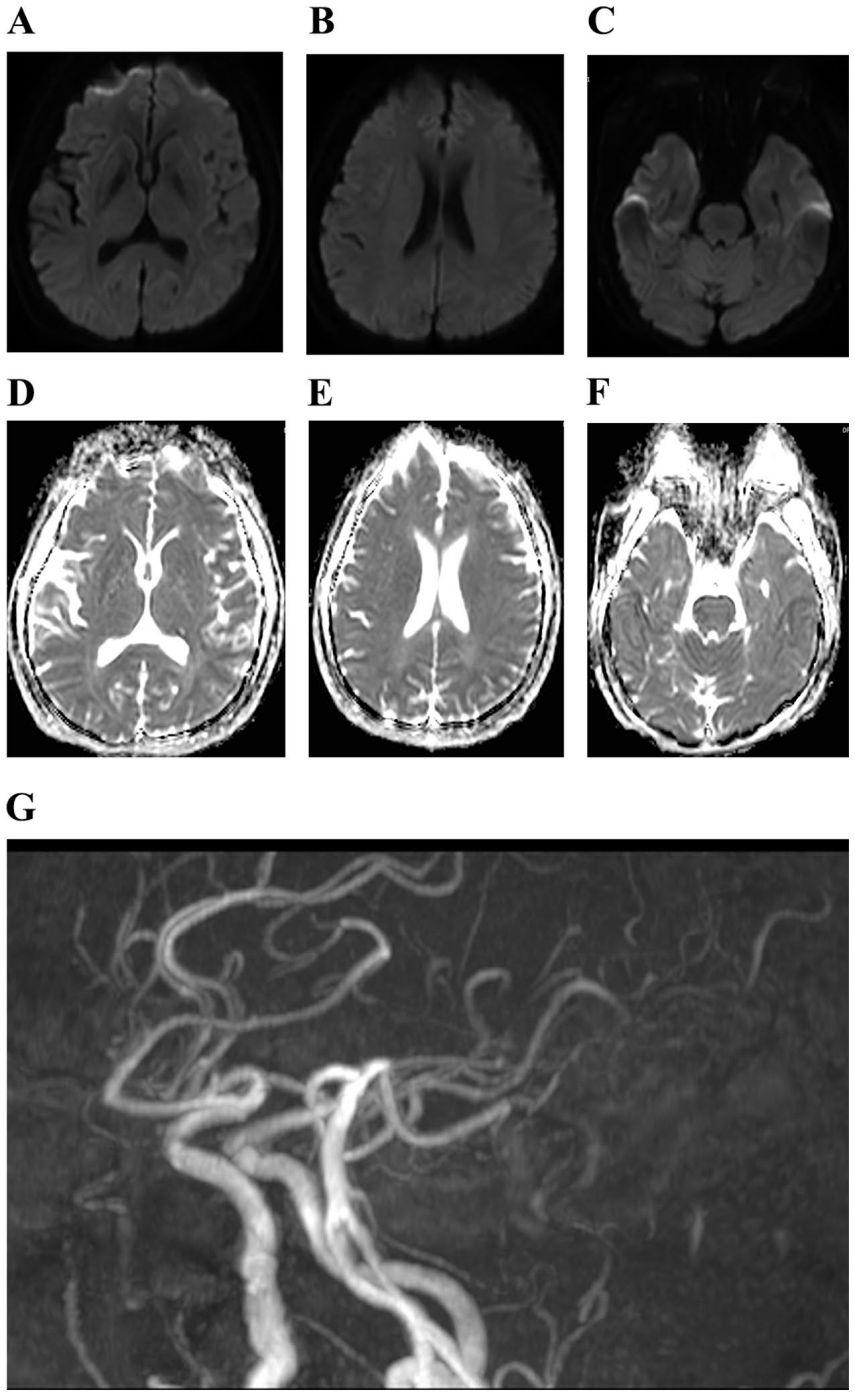
**(A–G)** Cranial magnetic resonance showed mild cerebral atrophy and cerebral atherosclerosis.

On admission, the patient had a body temperature of 38.8 °C, a heart rate of 112 beats per minute, a respiration rate of 20 beats per minute, and a blood pressure of 151/76 mmHg. Cardiopulmonary and abdominal examinations did not show any obvious abnormalities. A scar was observed between the index finger and thumb on the back of the right hand. Neurological examination showed clear mentation, articulation, and normal gross tests of higher neurological function. Both pupils were equal in size and round, with a diameter of about 3.0 mm, sensitive to light. Both eyes had adequate movement in all directions. The extension of the tongue showed centered teeth and normal pharyngeal reflexes. The muscle tone in the extremities was normal. The muscle strength of the left limb, right lower limb, and proximal part of the right upper limb was normal; however, the distal right upper limb was grade 0. The Babinski sign was negative, and meningeal irritation was also negative. Laboratory tests revealed the following blood routine indicators: white blood cell count at 9.39 × 10^9^/L, neutrophils at 7.19 × 10^9^/L, monocytes at 0.99 × 10^9^/L, eosinophils at 0.00 × 10^9^/L. The percentage of neutrophils, lymphocytes, and monocytes in the blood were 76.6, 12.8, and 10.5%. No abnormalities were detected in coagulation function, procalcitonin, liver function, renal function, electrolytes, cardiac enzymes, blood cultures, syphilis antibodies, viral hepatitis C antibodies, and human immunodeficiency virus antibodies. A CT scan of the lungs indicated no abnormality. The patient was prescribed enteric-coated aspirin tablets, clopidogrel tablets for antiplatelet aggregation, and statins for lipid regulation. Following admission to the hospital, the patient exhibited a progressive decline in muscle strength. The strength of the proximal muscle in the right lower and right upper extremity was assessed as normal, while the distal muscle strength of the right upper extremity was rated at grade 0. Proximal muscle strength of the left upper extremity was evaluated at grade 4, and its distal strength recorded as grade 0. The left Babinski’s sign was positive, while the right Babinski’s sign was negative. Lumbar puncture showed a CSF pressure of 300 mmH_2_O; the CSF nucleated cell counts was 25 × 10^6^/L, of which 0.92% were single nucleated cells and 0.08% were multiple nucleated cells. The CSF protein concentration was 571 mg/L. Rabies virus sequences were detected by CSF metagenomics sequencing. The mNGS employed the Probe-Capture Metagenomics method, and the total number of reads detected was 875,729. Finally, nine rabies virus reads were identified through bioinformatic analysis (Supplementary Table 1). Cranial magnetic resonance imaging, diffusion tensor imaging, and magnetic resonance angiography showed no abnormalities (Figure 3). On November 9, 2023, the patient suffered sudden respiratory cardiac arrest and fell into a deep coma, and was immediately given transoral intubation balloon assisted ventilation. The patient died on November 11, 2023, after an unsuccessful rescue effort. The chronological timeline of clinical events in the present case is shown in Table 1. The patient did not exhibit characteristic hydrophobia during the course of the disease. The patient was ultimately diagnosed with paralytic rabies.

**Table 1:** Chronological timeline of clinical events in the present case.

Day	Date (2023)	Event
0	14 October	Right hand bite by unvaccinated domestic dog (class III exposure). Immediate initiation of the “2-1-1” vaccine program, but no rabies passive immunization preparations
23	06 November	Numbness and weakness of the right upper limb appeared
24	07 November	Symptom aggravation
25	08 November	Hospitalization
26	09 November	Lumbar puncture was performed in the morning and CSF was sent for mNGS testing. Rapid deterioration in the afternoon with respiratory arrest and tracheal intubation
28	11 November	Patient death

CSF, cerebrospinal fluid; mNGS, metagenomics next-generation sequencing.

## 3 Discussion

Rabies is an acute zoonotic infectious disease caused by infection with the rabies Lyssavirus genus virus. It is recognized as the most lethal brain disease known so far. Most cases are transmitted through saliva carrying the virus, usually through the bite of an infected animal. However, transmission can also occur through other approaches, such as scratches and, in rare instances, through organ transplants and other routes (7). Once the disease onset occurs, the mortality rate is almost 100%, and effective treatment options remain insufficient (1, 2).

The natural progression of rabies is a continuous process, with clinical manifestations of the disease can be divided into the following phases: (1) Incubation period: the majority of cases exhibit an incubation period of 13 months, with rare cases occurring within 1 week or extending beyond 1 year. (2) Prodromal phase: manifested by symptoms such as unexplained fear, anxiety, agitation, irritability, nervousness, insomnia, or depression, etc., and usually accompanied with atypical symptoms such as malaise, anorexia, fatigue, headache, and fever. This phase usually lasts for 2 to 10 days. (3) Acute neurological symptomatic phase: divided into manic and paralyzed types, usually lasting for 1 to 3 days. Bipolar cases account for 80% of rabies, characterized by extreme fear, fear of water, fear of wind, pharyngeal muscle spasm, dyspnea, difficulties in urination and defecation, along with excessive sweating and salivation. Paralytic cases are characterized by high fever, headache, vomiting, and pain at the bite site, followed by limb weakness, abdominal distension, ataxia, and incontinence, without typical euphoria or hydrophobia. (4) Paralytic period: the phase when the case gradually transitions into a calm state after the acute neurological symptoms. During this time, spasms cease, the case gradually becomes quiet, and flaccid paralysis occurs, particularly in the limbs. The phase usually lasts from 6 to 18 h. (5) Death: patients die of respiratory and cardiac arrest shortly after the paralytic phase. Death often occurs due to respiratory and cardiac failure 7 to 10 days after the initial clinical signs. In this case, the patient experienced weakness and numbness in the distal part of the right upper limb, which progressively worsened accompanied by neck and shoulder pain, fever, and a peak body temperature of 38.8 °C. The patient had an incubation period of 23 days, and the prodromal symptoms were also atypical. There were no typical signs of extreme fear, hydrophobia, wind fear, spasms of pharyngeal muscle dyspnea, dysuria, dyspareunia, or excessive sweating and salivation, nor was there a period of acute neurological symptoms. The distal weakness and numbness progressively worsened to respiratory arrest, deep coma, and ultimately death. The total duration from latency to death was 28 days, while the total duration from the onset of prodromal symptoms to death was 5 days.

Encephalitic rabies (80% of cases) and paralytic rabies (about 20% of cases) are the two clinical forms of rabies manifested by central nervous system involvement (8). The more common form is encephalitic rabies, which occurs in about 80% of patients, with 50–80% of these patients displaying typical symptoms characteristic of rabies, such as hydrophobia and aerophobia (9). Encephalitic rabies usually progresses to severe flaccid paralysis, coma, and death due to multiorgan failure, whereas paralytic rabies exhibits significant muscle weakness during early stages of the disease. The rabies virus demonstrates a strong neurotropic effect on the anterior horn of the spinal cord and brainstem nuclei (10). Similar to middle cerebral artery or lacunar infarction, the early invasion of the corticospinal and spinothalamic tracts by the rabies virus can result in immediate weakness and sensory loss in the contralateral limb, which might be sometimes misinterpreted as a stroke (11). Mohindra et al. (12) reported a case of histopathologically confirmed rabies, in which the symptomatic episodes included recurrent instances of spontaneous inappropriate ejaculation. Park et al. (13) described a case of rabies involving cardiac complications due to rabies virus infection, which manifested as an acute ST-segment elevation myocardial infarction based on electrocardiographic, laboratory, and examination findings. Soler-Rangel et al. (14) reported a case initially diagnosed as acute adult respiratory distress syndrome (ARDS). The precise diagnosis was postponed due to the presence of initial extra-neurological manifestations and late reporting of rabies exposure, which was ultimately confirmed by a positive rabies virus immunofluorescence test. Chaudhary et al. (15) reported a case involving a 52-year-old man who was admitted to the hospital due to sudden weakness in his left arm. The patient was initially suspected to have Guillain–Barré syndrome (GBS), and did not receive intravenous immunoglobulin treatment, and unfortunately died on the day 24 after his initial visit. A subsequent follow-up reveled a history of exposure, leading to a confirmed diagnosis of rabies encephalitis through pathology and imaging.

Rabies needs to be diagnosed in the context of the epidemiologic history of the case, clinical manifestations, and laboratory findings. A clinical case is diagnosed when there is an epidemiologic history consistent with clinical signs of manic or paralytic rabies. Rabies is easier to diagnose when it manifests in its classical form. However, when it manifests as paralysis, the diagnosis is frequently delayed due to clinical symptoms that can be confused with other acute paralytic diseases such as GBS or acute disseminated encephalomyelitis (ADEM) (15). Nevertheless, the diagnosis of rabies can be confirmed when any of the laboratory tests are positive, or when combined with imaging and electroencephalography (EEG). Pin et al. (16) reported a case of a 59-year-old male patient who had a history of dog bites and unregulated wound management. The patient displayed atypical clinical symptoms at the onset and multiple brain magnetic resonance imaging scans indicated no abnormalities raising suspicion of an intracranial infection. Initially, the patient tested negative for rabies virus antibodies, and attempts to rabies virus from neck skin biopsy and saliva specimens, as well as the detection of viral antigen and the amplification of viral nucleic acid were unsuccessful. Ultimately, the patient was diagnosed with human rabies through mNGS. Once rabies manifests, the fatality rate is almost 100%. However, safe and effective RABV vaccines have been available for decades to provide a protective neutralizing antibody response for pre-immunization and post-exposure prophylaxis (17, 18). The administration of inactivated rabies vaccines, whether administered prior to or following exposure, in conjunction with anti-rabies immune globulin, represents the most effective strategy for preventing the development of clinical rabies and human mortality (19). In this instance, the short incubation period underscores the importance of appropriated post-exposure prophylaxis. The failure to administer anti-rabies immunoglobulin or serum at the time of exposure was a significant omission, which may have contributed to the inability to prevent the onset of the disease.

In this particular case, rabies was not diagnosed at the time of admission due to the patient’s stroke-like onset, and the absence of typical clinical signs associated with rabies. Furthermore, the family did not promptly disclose any history of domestic dog bites. There were no cerebrospinal fluid or cranial magnetic resonance imaging (MRI) features indicative of encephalitis rabies. In this case, no rabies virus was detected in the CSF of the patient using polymerase chain reaction (PCR), and the diagnosis of paralytic rabies was confirmed after sequencing of the cerebrospinal fluid macro-genome, suggesting that mNGS technology can effectively assist in the diagnosing paralytic rabies. While this case demonstrates the feasibility of detecting rabies virus RNA using mNGS, further prospective studies are needed to evaluate the sensitivity and specificity of this approach in a larger sample size.

On the morning of the second day of hospitalization, the patient underwent a lumbar puncture. Theoretically, the diagnostic delay might have been reduced if CSF had been collected earlier. Nevertheless, early confirmation would not have changed the ultimate prognosis, as neurological symptoms typically result in death. However, prompt identification through mNGS highlights the importance of timely CSF sample collection in suspected atypical rabies cases, as it offers essential information for instant contact tracing and risk assessment.

The direct fluorescent antibody (DFA) test employs fluorescence microscopy and high-quality antibodies, and it requires skin or brain tissues for the diagnosis of rabies. This test is prone to false negatives when specimens have low viral loads and is not applicable for detecting CSF virus *in vivo*. In the early stages of paralytic rabies or when CSF virus levels are extremely low, the polymerase chain reaction (PCR), which is sensitive to genotypic variation and requires pre-designed primers, demonstrates a low detection rate. In contrast, mNGS does not require prediction of target sequences and can detect all pathogens in an unbiased manner. In this case, nine rabies virus reads (>99% homology) were identified in a single test. Furthermore, mNGS can concurrently rule out immune-mediated disorders and other central nervous system infections while offering a differential diagnosis for atypical patients at molecular level.

In addition, mNGS technology is useful in regions with a high-prevalence of rabies, particularly where the detection capabilities of conventional DFA or PCR tests are constrained. Its unbiased, culture-free process allows local laboratories to identify rabies viruses directly from CSF or saliva, facilitating contact tracing, risk assessment, and targeted post-exposure prophylaxis. In locations lacking a dedicated rabies reference center, a centralized mNGS center can serve as a surveillance platform to simultaneously monitor viral diversity and detect new rabies virus variants without the need for additional assay development. Cost-effectiveness analyses and training programs are essential before the integration of this technology into routine public health practice.

## 4 Conclusion

The diagnosis of manic rabies relies on a clear history and typical signs of excitability and hydrophobia, such as extreme fear, hydrophobia, wind fear, pharyngeal muscle spasms, dyspnea, dysuria, dyspareunia, and excessive sweating and salivation. However, paralytic rabies, without the typical excitatory phase and hydrophobia, is often confused with other diseases, and the diagnosis is often delayed. Timely vaccination and passive immunotherapy are crucial, especially when the incubation period is short. High-throughput sequencing and the subsequent analysis of metagenomic data may provide new technological options for rapid, reliable, cost-effective, and sensitive diagnosis of rabies.

## Data Availability

Data not readily available because of ethical and privacy restrictions. Requests to access the datasets should be directed to the corresponding authors.
