# Supplementary material for: Application of metagenomics sequencing to diagnose paralytic rabies with stroke-like onset: a case report
Source: Front Med (Lausanne). 2025 Aug 26;12:1639262. doi: 10.3389/fmed.2025.1639262 (PMC12417114; doi:10.3389/fmed.2025.1639262)
Supplement: Supplementary file 1 [file Table_1.docx]

Table 1 Nine reads have been identified through bioinformatic analysis

|  | Read identifier | Nucleic acid sequence fragments |
| --- | --- | --- |
| 1 | FT100030277L1C001R00202300795 | GCGACTCCTTGCAAAAGACCATCCCTTAAATCCTTATATC |
| 2 | FT100030277L1C004R00202246706 | ACTTGTGGGACAACAGGAGGGGGGAATCCTAGGGTTTCAG |
| 3 | FT100030277L1C003R00400686703 | ATCCTAGGGTTTCAGTGTCTGTACTCCCGTCCTTCGATCA |
| 4 | FT100030277L1C001R00101906403 | AGACCCCTCAGATCTTTGAGTTTCCAGATGTGTCAAAAAG |
| 5 | FT100030277L1C001R00301964342 | CATCAGAGATACTGGATGACAAGTCACACTCTTTCACCAG |
| 6 | FT100030277L1C004R00501331895 | CGACCCAGTTGTTCCATGCCTGGGAAAAGGTCACCAATGT |
| 7 | FT100030277L1C008R00400844588 | AAGCTGTGAGGACAAGTCATCTCTTCTACATTTCGAGTCC |
| 8 | FT100030277L1C001R00301195510 | CAAATATTCGAAATCCCTGAATCCATGGATCCATCAGAGA |
| 9 | FT100030277L1C003R00101955499 | CATCTAAAACCCGGAGATTTTGAATCTCTAAGTGGTAGGG |
